# Methodology of the health economic evaluation of the Feel4Diabetes-study

**DOI:** 10.1186/s12902-019-0471-3

**Published:** 2020-03-12

**Authors:** Ruben Willems, Lore Pil, Christina-Paulina Lambrinou, Jemina Kivelä, Katja Wikström, Esther M. Gonzalez-Gil, Pilar De Miguel-Etayo, Anna Nánási, Csilla Semánová, Vicky Van Stappen, Greet Cardon, Kaloyan Tsochev, Violeta Iotova, Nevena Chakarova, Konstantinos Makrilakis, George Dafoulas, Patrick Timpel, Peter Schwarz, Yannis Manios, Lieven Annemans, on behalf of the Feel4Diabetes research group

**Affiliations:** 1 Department of Public Health and Primary Care, Ghent University, Corneel Heymanslaan 10, Entrance 42 – Floor 4, 9000 Ghent, Belgium; 2 Department of Nutrition and Dietetics, Harokopio University, 70 El Venizelou Ave, 176 71 Kallithea, Athens, Greece; 3 Department of Public Health Solutions, National Institute for Health and Welfare, Mannerheimintie 166, 00271 Helsinki, Finland; 4 Growth, Exercise, Nutrition and Development (GENUD) Research Group, University of Zaragoza, 50009 Zaragoza, Spain; 5 Institute of Nutrition and Food Technology, Center of Biomedical Research, University of Granada, Granada, Spain; 6 Instituto Agroalimentario de Aragon (IA2), Zaragoza, Spain; 7 Instituto de Investigacion Sanitaria Aragón (IIS Aragon), University of Zaragoza, Zaragoza, Spain; 8 Centro de Investigacion Biomedica en Red de Fisiopatologia de la Obesidad y Nutricion (CIBERObn), University of Zaragoza, Zaragoza, Spain; 9 Department of Family and Occupational Medicine, University of Debrecen, Debrecen, 400 Hungary; 10 Department of Movement and Sports Sciences, Ghent University, Campus Dunant, Watersportlaan 2, 9000 Ghent, Belgium; 11 Department of Paediatrics, Medical University Varna, 1 Hr. Smirnenski Blvd, 9010 Varna, Bulgaria; 12 Department of Diabetology, Clinical Center of Endocrinology, Medical University Sofia, Sofia, Bulgaria; 13 National and Kapodistrian University of Athens, 17 Ag. Thoma St, 11527 Athens, Greece; 14 Department for Precention and Care of Diabetes, Technische Universität Dresden, Fetscherstraße 74, 01307 Dresden, Germany; 15 Paul Langerhans Institute Dresden of the Helmholtz Center Munich at University Hospital and Faculty of Medicine, Technische Universitat Dresden, Dresden, Germany; 16 German Center for Diabetes Research (DZD e.V.), Neuherberg, Germany

**Keywords:** Health economics, Type 2 diabetes mellitus, Lifestyle, Intervention, Vulnerable group

## Abstract

**Background:**

The clinical and economic burden of type 2 diabetes mellitus on society is rising. Effective and efficient preventive measures may stop the increasing prevalence, given that type 2 diabetes mellitus is mainly a lifestyle-driven disease. The Feel4Diabetes-study aimed to tackle unhealthy lifestyle (unhealthy diet, lack of physical activity, sedentary behaviour, and excess weight) of families with a child in the first grades of elementary school. These schools were located in regions with a relatively low socio-economic status in Belgium, Bulgaria, Finland, Greece, Hungary and Spain. Special attention was paid to families with a high risk of developing type 2 diabetes mellitus.

**Methods:**

The aim of this paper is to describe the detailed methodology of the intervention’s cost-effectiveness analysis. Based on the health economic evaluation of the Toybox-study, both a decision analytic part and a Markov model have been designed to assess the long-term (time horizon of 70 year with one-year cycles) intervention’s value for money. Data sources used for the calculation of health state incidences, transition probabilities between health states, health state costs, and health state utilities are listed. Intervention-related costs were collected by questionnaires and diaries, and attributed to either all families or high risk families only.

**Conclusions:**

The optimal use of limited resources is pivotal. The future results of the health economic evaluation of the Feel4Diabetes-study will contribute to the efficient use of those resources.

## Background

Prevalence of Diabetes Mellitus is on the rise. While 50 years ago, the worldwide prevalence of patients with diabetes was estimated to be approximately 30 million [1], the latest estimates of the International Diabetes Federation (IDF) go up to 415 million patients with diabetes aged 20 to 79, accounting for a global healthcare burden of 673 billion US dollars. Without improved preventive measures, the prevalence is expected to rise exponentially with another 50% by 2040. Over 10% of the global population will suffer from diabetes [2].

Type 2 diabetes mellitus (T2DM) is mainly a lifestyle-driven disease [3], with sedentary behaviour, lack of physical activity, unhealthy diet, and excess weight among the most important risk factors [4]. The Feel4Diabetes-study aimed to tackle T2DM in elementary school children and their parents through intensive lifestyle modifications [5]. The British National Institute for Health and Care Excellence (NICE) guidelines recommend to focus lifestyle interventions on populations at risk [6], such as low-to-middle-income countries (LMICs) where 80% of all T2DM patients live [7], and regions in high-income countries (HICs) with high unemployment rates (31% increased risk) or low average education levels (41% increased risk) [8]. Hence, the Feel4Diabetes-study targeted low socioeconomic status (SES) communities, and special attention is paid to families at increased risk to develop T2DM within those low SES communities [5]. All adults were screened with the Finnish Diabetes Risk Score (FINDRISC) questionnaire (a short questionnaire assessing age, body mass index (BMI), waist circumference, lifestyle, and medical history) to differentiate high risk families (HRF) from low risk families (LRF) [9]. Extra measures, such as counseling sessions, were taken to mitigate the risk level in the HRF. The intervention group was compared to a control group in different schools. Control schools were asked to continue with their standard curriculum, but HRF in the control group received general advice for a healthy and active lifestyle in a one-hour session. The total study sample comprised of 6450 and 5743 families, of which 1273 and 957 were HRF, in the respective intervention and control group. Participating children were on average 8.2 years old. About 90 and 78% of the maternal and paternal parent were younger than 45 years old. A detailed description of the Feel4Diabetes-study and the participating population can be found in Manios et al. [5].

A health economic evaluation informs healthcare policy makers on the value for money of alternative healthcare services across disciplines and countries. This paper outlines the design and the data input used for the health economic evaluation of the Feel4Diabetes-study. The Feel4Diabetes-study had been implemented in six participating countries. Belgium and Finland represented the HICs, Greece and Spain the high-income countries under austerity measures, and Hungary and Bulgaria the LMICs. Low SES regions had been determined in HICs as described elsewhere [5]. All regions in Hungary and Bulgaria were defined as low SES regions. Total intervention time covered two years, starting in September 2016 [5].

## Methods

Our health economic model is based on the model by Pil et al. [10], and has been modified with respect to the intervention’s objectives and target population’s characteristics. The original model was developed to assess the Toybox-intervention [11], the aim of which was to tackle obesity. Pil et al. described an indirect method to conduct a health economic evaluation of measures preventing non-communicable diseases from childhood on. Changes in energy balance-related behaviours (EBRBs) were used as predictors for weight loss, eventually leading to a reduction in disease prevalence. However, the main objective of the Feel4Diabetes-study was to reduce the risk of T2DM. Since the population in the study was young and the follow-up time only 2 years, instead of measuring the incidence of T2DM, the risk markers of T2DM were measured. Excess weight is one of the best risk markers, so BMI is the main surrogate marker for T2DM in this study, although there is no one-on-one relation. Still, a modified version of the original model was considered to be the best possible option in meeting our study’s objective. A comprehensive justification for our choice to select this model can be found in the discussion section.

### Structure of the health economic model

The health economic model consists of a decision analytic part and a Markov model. The intervention cost will be weighed against the intervention’s health benefits in both the children and their parents since Feel4Diabetes is a holistic school- and community based intervention impacting the life of the whole family.

### Decision analytic part

The decision analytic part of the health economic model will run based on (i) intervention-driven relative risk reductions (RRR) in overweight and obesity and/or (ii) RRR in EBRBs, with the former option getting priority. The first option is to classify participants on weight status (based on BMI), resulting in three groups: normal weight (BMI < 25), overweight (BMI 25–30) and obesity (BMI > 30). The Feel4Diabetes-study will result in different weight status distributions between the intervention and control group. The second option is to conduct a health economic evaluation based on an RRR in eight targeted, mediating EBRBs (Table 1), indirectly affecting the weight status distribution. This approach may be preferred over the direct BMI-approach because it can take years before a full decrease in bodyweight after a change in lifestyle can be observed [21]. We were able to directly derive the relative risk (RR) of EBRBs on obesity/overweight from studies [19, 20] or to calculate the RR if the risk in the control group was reported [12, 14, 15, 17, 18]. In the event that an RR could not be derived, a conservative RR was estimated based on the odds ratio (OR) and the risk in the total sample [13, 15, 16]. In children, the literature seems to be inconclusive on the association between the consumption of fruits and berries, vegetables and sweets on the one hand, and weight status on the other hand. In adults, the literature seems to be inconclusive on the association between the consumption of water and sweets on the one hand, and weight status on the other hand. Hence the exclusion of the aforementioned EBRBs from analyses (Table 1).

**Table 1:** EBRBs and relative risk on overweight/obesity

	Children			Adults		
EBRBs	behaviour	Relative risk on overweight/obesity	reference	behaviour	Relative risk on overweight/obesity	reference
Water consumption	1.1 glass of water per day difference	1.33	[12]		inconclusive literature	
Fruits and berries		inconclusive literature		3.13 daily servings difference	1.23^Ɨ^ (overweight)1.25^Ɨ^ (obesity)	[13]
Vegetables		inconclusive literature		3.13 daily servings difference	1.19^Ɨ^ (overweight)1.15^Ɨ^ (obesity)	[13]
Screen time	> 4 h per day	2.00	[14]	> 21 h per week	1.38	[15]
Sweets		inconclusive literature			inconclusive literature	
Sugar-sweetened beverages	> 1 sugary drink per day	1.22^Ɨ^	[16]	> 1 soft drink per day	1.30	[17]
Daily physical activity	< 60 min per day	1.35	[18]	< 5 days per week 30 min	1.07^Ɨ^	[15]
Breakfast pattern	daily breakfast taking	3.03 (overweight); 2.13 (obesity)	[19]	daily breakfast taking	1.19	[20]

Ɨ: conservative estimates of relative risks, derived from odds ratio and prevalence in the control group.

### Markov model

**Fig. 1:**
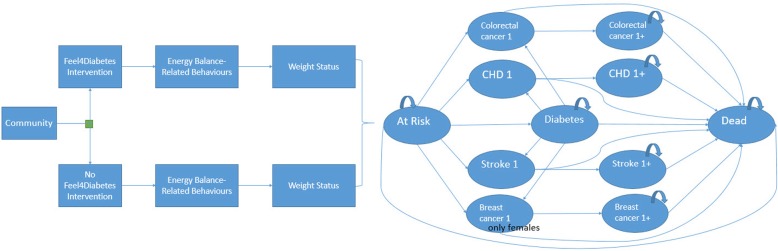
Markov model with 11 health states

T2DM incidence and mortality in young adulthood is low to negligible [4]. Therefore, the subsequent Markov model starts at age 30 and stops after 70 one-year cycles, enabling us to capture long-term effects and costs (Fig. 1). Both the intervention effect on the targeted children and their parents are incorporated in the model. The adult population is split up in six age groups (< 30, 30–34, 35–39, 40–44, 45–49, > 50). Parents who are younger than 30 and older than 50 start in the model at the respective age of 30 and 50. The proportion in, for instance, the age group 30–34 is distributed evenly over the years. Children’s entry in the Markov model is less straightforward. As in Pil et al. [10], we used the tracking study of Venn et al. [22] to extrapolate the child’s current weight status to his/her weight status at adult age (the start of the Markov model).

The 11 health states are included in the health economic model (Fig. 1): at risk, diabetes, stroke, coronary heart disease (CHD), colorectal cancer (CRC), breast cancer (BC), and death. A differentiation is made between the first year after diagnosis (e.g. CHD1) and follow-up years (e.g. CHD1+) in stroke, CHD, CRC, and BC as utility levels and costs appeared to be significantly different. Only females can make the transition to the health state BC. The model is a simplification of reality since other comorbidities are not considered. The entire cohort starts in the ‘at risk health state’, with a distribution of weight status. Transition probabilities are a function of the underlying weight status distribution, which is different between the intervention and the control group as a result of the intervention.

### Main health economic outcome

The incremental-cost-effectiveness ratio (ICER) is the main health economic outcome and can be expressed as costs per Quality Adjusted Life Year (QALY) gained. A QALY is a measurement that incorporates both the quantity (the number of years lived in a certain health state) and quality of life (a score of 0 represents death and 1 represents perfect health). The ICER is the ratio between the difference in costs and the difference in QALYs between the intervention cohort and the control cohort:

$$ ICER=\frac{\mathrm{COSTintervention}-\mathrm{COSTcontrol}}{\mathrm{QALYintervention}-\mathrm{QALYcontrol}} $$

The model was developed with Microsoft® Excel 2016 (Microsoft Corporation, Redmond, WA, USA).

### Clinical data input

#### Epidemiological data

Country-, gender- and age-specific weight status prevalence were derived from Eurostat [23]. Weight status prevalence in all countries, except Bulgaria and Hungary, were adjusted for SES. Since low SES regions were defined differently across countries [5], we chose to adjust for SES in a conservative way: we calculated the RR for excess weight in the lower educated half of the population. Table 2 shows how overweight and obesity prevail more often in low SES groups, except in the Finnish population [24].

**Table 2:** Relative risk to be overweight and obese in the lower educated half of the adult population, compared with the total adult population

		Belgium	Finland	Greece	Spain
Men	Overweight	1.05	0.97	1.07	1.07
	Obese	1.12	1.03	1.30	1.30
Women	Overweight	1.24	1.04	1.33	1.33
	Obese	1.39	0.89	1.46	1.46

#### Transition probabilities

- Transition probabilities from the at risk state to disease states in the control group were derived from European databases and international publications [10, 25–30]. The transition probabilities in the intervention group were adjusted for the change in weight status as a result from the Feel4Diabetes intervention. For instance, overweight and obese men have respectively 125 and 450% more risk to develop T2DM (Table 3) [31]. Missing data were imputed by calculating the diseases’ total incidence ratio between countries with Belgium as reference country.
- Transition probabilities from the diabetes state to other disease states: patients with diabetes are at risk to develop other diseases. Compared to healthy counterparts, the RR to develop comorbidities ranges from 1.23 for BC to 2.19 for CHD (Table 3) [32–34].
- Transition probabilities to the death state: country-, gender- and age-specific all-cause mortality rates were derived from Eurostat, the World Health Organization and the Belgian mortality table [35–37]. These rates were multiplied with the relative mortality risk for patients with diabetes, to obtain the mortality risk for the health state ‘diabetes’ (Table 3) [38]. Belgian cancer mortality rates were obtained from the Belgian Cancer Registry [39, 40]. Dutch data were used to estimate mortality in CHD and stroke [41] since Belgian data were not available. Missing data were imputed by calculating the diseases’ total mortality ratio between countries with Belgium as reference country.
- Transition probabilities from disease state (e.g. BC) to the follow-up disease state (e.g. BC1): the transition probability is 100% minus the transition probability to the death state.

**Table 3:** Relative risk values used in the health economic evaluation of F4D. Overweight and obese men/women compared to normal weight. Diabetic men/women compared to healthy counterparts

		All-cause mortality				CHD			Stroke		Diabetes			BC		CRC	
	Age	< 50	50–59	60–69	70+	< 55	< 65	65+	< 65	65+	<60y	60-74y	75+	< 50	50+	< 45	45+
At Risk Men	Overweight	1.20	1.20	1.19	1.18	1.35	1.35	1.25	1.20	1.15	2.25	2.15	2.13	–	–	1.20	1.18
	Obese	1.55	1.54	1.52	1.50	2.00	2.00	1.70	1.50	1.38	5.50	5.14	5.05	–	–	1.40	1.36
At Risk Women	Overweight	1.15	1.15	1.14	1.14	1.35	1.35	1.25	1.20	1.15	2.30	2.20	2.17	1.00	1.12	1.08	1.07
	Obese	1.50	1.49	1.48	1.45	2.00	2.00	1.70	1.55	1.41	7.00	6.52	6.40	1.00	1.12	1.10	1.09
Diabetic Men		1.57	1.57	1.57	1.57	2.19	1.43	1.33	1.83	1.83	–	–	–	1.23	1.23	1.26	1.26
Diabetic Women		2.00	2.00	2.00	2.00	2.19	1.43	1.33	2.28	2.28	–	–	–	1.23	1.23	1.26	1.26

BC: breast cancer; CHD: coronary heart disease; CRC: colorectal cancer

#### Utilities

Health state-specific utility values to calculate QALYs were derived from international literature [42–46] and can be found in Additional file 1: Table S1. More specifically, a utility decrement was calculated for each disease. Country-specific publications were applied where possible, but we frequently had to extrapolate data from other European countries [44, 47–59]. For instance, since no Bulgarian data were available, Bulgarian utilities were assumed equal to Hungarian utilities. In our model, we differentiated between the first year after diagnosis and the follow-up years. Patients may experience an improved quality of life in the follow-up years but they risk a relapse, which we took into account when calculating the health state utilities [60–63].

### Cost data input

All costs are converted to the euro currency value of the year 2016 if necessary. The health economic evaluation considers two types of costs: disease state-related costs and intervention costs. A societal perspective is chosen to incorporate both direct (medical) costs and indirect costs associated with productivity loss.

#### Disease state costs

Direct, country-specific, annual disease costs were derived from published literature [59, 64–79].

The indirect costs of T2DM, BC and CHD were calculated by multiplying the direct costs with respectively 0.91, 0.71 and 0.8 [80–82]. The indirect cost of stroke equals the direct cost [66, 82]. The country-specific indirect costs of CRC were extrapolated from Finnish data. A ratio of direct/indirect CRC costs was calculated based on Farkkila et al., and applied on other countries [68].

The indirect cost related to death was calculated with a friction cost period of 160 days [83]. The actual hours worked within this time interval was multiplied with the average productivity cost per hour [84, 85]. These costs were only applied on participants between the age of 30 and 64, and were adjusted for the country-specific unemployment rate and for the principle of labour time elasticity, which states that production drops 8% when labour time drops 10% [83, 86].

To account for T2DM as a comorbidity in other disease states, 17.91, 19.80, 23.75 and 38.24% of the cost of T2DM was added to CHD, stroke, BC and CRC, respectively [87–89]. Costs in follow-up states were adjusted for potential relapse [60–63]. Costs were stratified for age (younger and older than 65 years) and an extra distinction was made between costs in T2DM patients younger and older than 55 years [90]. Missing data were imputed based on countries’ health expenditure per capita [91, 92] with Belgium as reference country.

Additional file 1: Table S2 shows the total costs per disease, stratified for age and country.

#### Intervention costs

Intervention costs can be attributed to the school-based component - which targets all children - or to the HRF component. Only those costs that would also be incurred in a future real-life implementation of the intervention were included in the evaluation. Therefore, costs attributable to the project planning, intervention material development or scientific evaluation were excluded. Costs attributable to the distribution and analysis of the EBRBs questionnaire were taken partly into account, as it contains the FINDRISC questionnaire [9] to classify families into LRF and HRF. Hence, it is part of the intervention model: these costs were attributed to the HRF component. Table 4 summarizes the intervention costs related to the school-based and HRF component.

**Table 4:** Intervention costs

	School-based component	High Risk Family component
Scientific Staff	· Time attributed to communication with schools, directors and teachers · Facilitation of the intervention (information distribution, feedback, problem-solving) · Delivery of intervention material· Delivering the teachers’ training session · Transportation costs	· Time attributed to communication with high-risk parents · Delivering the HRF group and individual sessions · Transportation costs
Community Stakeholders and NGO’s	· Extra time spending due to the study · Extra incurred costs due to the study	
High Risk Families		· Transportation cost to the counseling sessions · Time spending at the counseling session· Incurred costs related to a changing lifestyle (e.g. gym subscription, training equipment, weight scale)
Teachers	· Travel time to the training session· Transportation cost to the training session · Time spent at the training session· Time spent for the implementation of the intervention before and after school time · Incurred costs related to the implementation of the intervention.	
Other	· Distribution cost and production cost of newsletters· Other intervention costs reported by the scientific staff (i.e. intervention material)	· Distribution, collection and analysis of the FINDRISC questionnaire· Costs related to the SMS intervention · Other intervention costs reported by the scientific staff (i.e. intervention material)

#### Intervention costs related to the school-based component

The school-based component focused on changes at school, at home and at the municipality level. First, a questionnaire collected the costs associated with teachers’ training (mode of transport, transportation time, training time). In addition, teachers were asked at the end of the first intervention year if they invested extra time, next to their regular labour time, or spent money due to the project. The same method was applied to collect the costs made by collaborating community stakeholders and non-governmental organizations. Time and costs spent at already existing community activities (and which are not extended due to the Feel4Diabetes-study) were not included as these costs were not incremental. Second, newsletters made families aware of opportunities to change their lifestyle in a healthy way. All children in a class received the newsletters, but not all children participated in the Feel4Diabetes-study. Therefore, we computed the accurate cost of producing and distributing the newsletters to participants. Third, monthly diaries assessed the time-investment of project staff (e.g. communication with schools, training the teachers, SMS-intervention), their transportation costs and miscellaneous costs.

#### Intervention costs related to the HRF component

The HRF component extended the school-based component by offering six group and individual counseling sessions to HRF parents during the first intervention year and a 7th session at the start of the second intervention year. HRF were asked to report all incurred lifestyle modification costs (e.g. gym costs, sport clothes, cooking books) and their transportation mode to the counseling sessions. The 7th counseling session introduced HRF to the SMS-intervention, which ran in the second intervention year [5]. A monthly questionnaire was filled in by the Finnish firm Extensive Life Oy (developer of the SMS-intervention) to collect all SMS-intervention-related costs. Some countries produced invoices due to country-specific modifications of the intervention. These invoices were used to validate reported costs.

Missing data in HRF and in teachers were imputed based on the average of available information in the country of interest. Kilometer refund in the case of transportation per car is a function of the countries’ unleaded 95 RON gasoline price (October 27th, 2017) and the official Belgian work-related kilometer refund in 2016/2017.

### Analysis

Results will be reported as QALYs and costs per 1000 boys or girls targeted, and stratified for the HRF-component and the all families (LRF and HRF) component. Effects are discounted at 1.50% and costs at 3%, conform Belgian Health Care Knowledge Centre’s guidelines. As mentioned above, the intervention effect in children has been extrapolated from childhood to the point in adulthood that they enter the Markov model. Thereby discounting already started in childhood, leading to very strong discounts of the long term health effects and costs. Tornado diagrams (one-way sensitivity analysis) will display ±30% uncertainty intervals surrounding included parameters. Furthermore, second order Monte Carlo simulation will capture parameter uncertainty by varying parameters all together. Included parameters in the sensitivity analyses will be annual cost and utilities of health states, health states’ annual incidence and mortality rates, effect of the intervention, intervention cost, and RRR (in weight status or EBRBs). Several scenario analyses (e.g. maximizing and minimalizing the discount rate) will be conducted to capture uncertainty regarding modeling assumptions. Costs, probabilities and RRR are modeled using a gamma distribution, a beta distribution and a lognormal distribution respectively [93]. Budget impact analysis will assess the scalability of the intervention. Only the intervention costs for the healthcare budget holder will be included (e.g. HRF’s transportation costs to the sessions will be excluded). The budget impact will be calculated for different time horizons between 1 and 30 years, with the intervention being implemented every three years. Only the avoided healthcare costs in the parents will be included as cost offsets (the long term avoided healthcare costs in children are excluded due to the extrapolation of the intervention effect in childhood to benefits in adulthood, i.e. beyond the time horizon of the budget impact analysis). We assume the size of the target population to be stable over time.

## Results

N/A.

## Discussion

The Feel4Diabetes-study consisted of a school-, community-, and family-based intervention targeting T2DM in low SES regions. Additional measures were taken to foster outcomes in HRF [5]. One of the project’s objectives was to conduct a health economic evaluation, as health policy makers are burdened with the task to make use of the restricted budget in an efficient way. The current paper describes the methodology on how the long-term cost-effectiveness of the Feel4Diabetes-study will be assessed.

A two-part health economic model, consisting of a decision analytic part and a Markov model, was designed based on the health economic model used to assess the Toybox-study [10, 11]. Although Toybox’s main focus was obesity while the Feel4Diabetes-study focused on T2DM, the newly designed model suits our aims the best. Weight status is in our model the mediator to reduce T2DM because the Feel4Diabetes-study aimed to tackle T2DM by targeting obesity and obesity-related metabolic risk factors [5]. Given that 90% of T2DM patients have excess weight [94], it is not surprisingly the single best T2DM incidence predictor, with an explanatory factor of at least 60% [95]. The same risk factors were targeted in Toybox. In fact, some intervention materials from Toybox were adapted to the specific needs in the Feel4Diabetes-study [5, 11]. Weight status is associated not only with diabetes but with a range of pathologies (e.g. CRC), resulting in 11 health states in our model. It was crucial to include these health states to not underestimate the intervention’s effect. Differentiating the diabetes health state by adding health problems such as nephropathy and foot ulcers/amputations [96, 97] would be appropriate if the intervention’s target population were patients with diabetes, contrary to the ‘at risk target population’ in the Feel4Diabetes-study. Moreover, extra assumptions would then have been made to assign country-specific costs and utilities to those health states. This would affect the model’s complexity and transparency significantly. However, it is important to find the right balance between specificity and complexity, i.e. transparency. Therefore, we chose to include a general diabetes health state as an intermediate endpoint, for which we were able to assign age- and country-specific costs and utilities.

Predicting the cost-effectiveness results at this point is speculative, though published literature may set the prospects. Li et al. [98] reviewed T2DM prevention programs focusing on combined diet and physical activity promotion in at-risk populations. There was a wide variety in delivery methods across the prevention programs: individual-based, group-based and mixed. All but two studies (out of 16 studies reporting ICERs) reported ICERs under the applied willingness-to-pay threshold. The studies in which the cost effectiveness exceeded the acceptable threshold were both individual-based. As described above, the Feel4Diabetes-study focused on low SES regions and HRF received both individual and group counseling sessions. The total intervention cost of the included studies in Li et al.’s review [98] varied based on whether healthcare professionals (such as physicians or nurses) or trained laypersons (such as lay health educators, or trained community health workers) delivered the intervention, with the latter being less costly. It was decided that in the Feel4Diabetes-study, in order to improve the potential cost-effectiveness of the study, the researchers (trained laypersons) would be trained to deliver the individual- and group-based sessions. Moreover, Li et al. [98] found that programs more cost-effective longer-term follow-up studies. Therefore, an SMS-intervention is designed to take place in the second year of the intervention to foster the outcomes obtained by the more intensive first-year intervention. A review-of-reviews supported the effectiveness claim of SMS-interventions in for instance diabetes self-management and weight loss but cost-effectiveness data was lacking [99]. However, a recent study found dominant results of SMS-interventions in the prevention of T2DM [100].

### Limitations

Every health economic model is a simplification of the reality. The current health economic evaluation is also limited by the study’s multi-country perspective. The accessibility of health and economic data differs across countries. Clinical data can be extrapolated across countries with caution but economic data is a function of country-specific characteristics [101]. Therefore, data imputation was inevitable. We were able to mitigate this limitation by consequently using Belgian data as starting point for the imputation process. It needs to be emphasized that even though clinical data extrapolation across countries is possible, this remains a limitation of the health economic evaluation. We applied clinical data extrapolation several times (e.g. Dutch CHD and stroke mortality rates extrapolated to the Belgian context). Furthermore, diaries and process evaluation questionnaires were used to collect cost data collection. Although templates were available, it is not unthinkable that researchers and participants might interpret questions differently. Moreover, one disadvantage of questionnaires is the recall bias, leading to less accurate input data. In addition, as already stated by Pil et al. [10], measurements such as waist circumference might be more valid predictors for T2DM than BMI. Waist circumference was only measured in adults in F4D. To keep the model manageable and to increase its uniformity, we decided to use BMI as the predictor. The fact that we had to use intermediate endpoints such as BMI, and we could not use measured hard endpoints such as T2DM prevalence is a major shortcoming of the health economic evaluation. Finally, we extrapolated the intervention effect on children to the adult age, which increases the uncertainty of the analysis significantly. Calculated endpoints are subject to the participants’ lifestyle as teenager and young adult. Appropriate and extensive sensitivity analysis will therefore be conducted.

## Conclusion

The Feel4Diabetes-study aims to tackle T2DM by weight status-related lifestyle modifications in populations at risk. As policy makers cannot fund all interventions that turn out to be effective, health economic evaluations have the advantage to contribute to the optimal use of the limited resources. The current paper describes the methodology behind the cost-effectiveness assessment of the Feel4Diabetes-study.

## Supplementary information

**Additional file 1: **Supplementary material can be found online. **Table S1.** Disease costs, stratified for age and country. **Table S2.** Disease-specific utility values, stratified for age and country.

## Data Availability

The health economic model is available from the corresponding author on reasonable request.

## Abbreviations

BC: Breast Cancer
BMI: Body Mass Index
CHD: Coronary Heart Disease
CRC: Colorectal Cancer
EBRB: Energy-Based Related Behaviours
HIC: High-Income Countries
HRF: High Risk Families
ICER: Incremental Cost-Effectiveness Ratio
IDF: International Diabetes Federation
LMIC: Low-to-Middle Income Countries
LRF: Low Risk Families
NICE: National Institute for Health and Care Excellence
QALY: Quality Adjusted Life Year
RR: Relative Risk
RRR: Relative Risk Reduction
SES: Socio-Economic Status
T2DM: Type 2 Diabetes Mellitus

## About this supplement

This article has been published as part of BMC Endocrine Disorders, Volume 20 Supplement 1, 2020: Designing, implementing and evaluating a community-based intervention to prevent diabetes in vulnerable families across Europe. The Feel4Diabetes-study. The full contents of the supplement are available at https://bmcendocrdisord.biomedcentral.com/articles/supplements/volume-20-supplement-1.
