# Supplementary material for: Methodology of the health economic evaluation of the Feel4Diabetes-study
Source: BMC Endocr Disord. 2020 Mar 12;20(Suppl 1):14. doi: 10.1186/s12902-019-0471-3 (PMC7066818; doi:10.1186/s12902-019-0471-3)
Supplement: Supplementary file 1 — Additional file 1: Supplementary material can be found online. Table S1. Disease costs, stratified for age and country. Table S2. Disease-specific utility values, stratified for age and country. [file 12902_2019_471_MOESM1_ESM.docx]

**Table S1. Disease-specific utility values, stratified for age and country [1-22].**

|  |  | | **Belgium** | **Finland** | **Greece** | **Spain** | **Hungary** | **Bulgaria** |
| --- | --- | --- | --- | --- | --- | --- | --- | --- |
| **At risk state utility** | | 30-39 | 0.85 | 0.91 | 0.92 | 0.94 | 0.89 | 0.89 |
|  | | 40-49 | 0.83 | 0.89 | 0.83 | 0.92 | 0.84 | 0.84 |
|  | | 50-59 | 0.80 | 0.85 | 0.82 | 0.86 | 0.78 | 0.78 |
|  | | 60-69 | 0.79 | 0.80 | 0.69 | 0.75 | 0.81 | 0.81 |
|  | | 70-74 | 0.79 | 0.78 | 0.65 | 0.67 | 0.80 | 0.80 |
|  | | 75+ | 0.69 | 0.63 | 0.65 | 0.67 | 0.78 | 0.78 |
| **Disease penalty** | | Diabetes | 0.20 | 0.17 | 0.16 | 0.18 | 0.22 | 0.22 |
|  | | Colorectal Cancer 1 | 0.27 | 0.29 | 0.23 | 0.34 | 0.29 | 0.29 |
|  | | Colorectal Cancer 1+ | 0.25 | 0.26 | 0.20 | 0.30 | 0.26 | 0.26 |
|  | | Breast Cancer 1 | 0.07 | 0.09 | 0.03 | 0.14 | 0.09 | 0.09 |
|  | | Breast Cancer 1+ | 0.04 | 0.06 | 0.00 | 0.07 | 0.06 | 0.06 |
|  | | Stroke 1 | 0.25 | 0.19 | 0.21 | 0.32 | 0.27 | 0.27 |
|  | | Stroke 1+ | 0.21 | 0.24 | 0.17 | 0.42 | 0.23 | 0.23 |
|  | | CHD 1 | 0.11 | 0.12 | 0.12 | 0.18 | 0.12 | 0.12 |
|  | | CHD 1+ | 0.01 | 0.15 | 0.06 | 0.17 | 0.14 | 0.07 |

**Table S2. Disease costs, stratified for age and country. Values in italic are imputed [18-41].**

|  | Belgium | Finland | Greece | Spain |  | Hungary | Bulgaria |
| --- | --- | --- | --- | --- | --- | --- | --- |
| **Total cost diabetes 30-54** | 5 170 € | 6 954 € | 1 817 € | 3 934 € |  | 4 335 € | 3 057 € |
| **Total cost diabetes 55-64** | 8 524 € | 11 466 € | 2 996 € | 6 486 € |  | 7 148 € | 5 041 € |
| **Total cost diabetes 65+** | 5 452 € | 6 210 € | 1 716 € | 3 648 € |  | 3 804 € | 2 704 € |
| **Total cost Colorectal cancer 1 (aged 30-64)** | 31 378 € | 35 048 € | *10 594 €* | *17 760 €* |  | *8 401 €* | *5 761 €* |
| **Total cost Colorectal cancer 1 (aged 65+)** | 23 641 € | 26 024 € | *8 141 €* | *13 413 €* |  | *5 856 €* | *4 025 €* |
| **Total cost Colorectal cancer 1+ (aged 30-64)** | 20 364 € | 21 180 € | *6 875 €* | *11 732 €* |  | *6 114 €* | *4 211 €* |
| **Total cost Colorectal cancer 1+ (aged 65+)** | 14 776 € | 14 936 € | *5 067 €* | *8 456 €* |  | *3 980 €* | *2 748 €* |
| **Total cost breast cancer 1 (aged 30-64)** | 26 622 € | 26 032 € | *8 798 €* | 28 288 € |  | *6 761 €* | *4 614 €* |
| **Total cost breast cancer 1 (aged 65+)** | 16 055 € | 15 557 € | *5 532 €* | 17 546 € |  | *3 927 €* | *2 698 €* |
| **Total cost breast cancer 1+ (aged 30-64)** | 16 879 € | *16 764 €* | *5 593 €* | 6 964 € |  | *4 699 €* | *3 221 €* |
| **Total cost breast cancer 1+ (65+)** | 10 181 € | *9 938 €* | *3 495 €* | 4 243 € |  | *2 685 €* | *1 851 €* |
| **Total cost stroke 1 (aged 30-64)** | 29 467 € | 32 147 € | 10 669 € | 14 723 € |  | 4 488 € | 3 929 € |
| **Total cost stroke 1 (aged 65+)** | 15 372 € | 16 705 € | 5 876 € | 7 965 € |  | 2 311 € | 2 036 € |
| **Total cost stroke 1+ (aged 30-64)** | 12 737 € | 7 332 € | 6 248 € | 6 394 € |  | 1 864 € | 1 495 € |
| **Total cost stroke 1+ (aged 65+)** | 6 712 € | 3 801 € | 3 434 € | 3 447 € |  | 949 € | 768 € |
| **Total cost CHD 1 (aged 30-64)** | 21 493 € | 19 116 € | 10 727 € | 11 573 € |  | *5 406 €* | 5 086 € |
| **Total cost CHD 1 (aged 65+)** | 12 360 € | 10 908 € | 6 466 € | 6 843 € |  | *3 015 €* | 2 867 € |
| **Total cost CHD 1+ (aged 30-64)** | 7 355 € | 4 170 € | 5 905 € | 4 358 € |  | *2 427 €* | 1 900 € |
| **Total cost CHD 1+ (aged 65+)** | 5 177 € | 3 339 € | 3 830 € | 3 147 € |  | *1 949 €* | 1 447 € |
| **Total indirect cost of death** | 21 321 € | 19 245 € | 10 134 € | 12 661 € |  | 5 126 € | 2 683 € |

**References**

1. Baji P, Brodsky, V, Rencz, F, Boncz, I, Gulacsi, L & Pentek, M. A magyar lakossag egeszsegi allapota 2000 es 2010 kozott [Health state of the Hungarian population between 2000-2010]. Orv Hetil. 2015;156(50):10.

2. Charafeddine R. Gezondheidsgerelateerde kwaliteit van leven [Health-related Quality of Life]. In: WIV-ISP, editor. Brussel: Van der Heyden J CR; 2014.

3. Saarni SI, Harkanen T, Sintonen H, Suvisaari J, Koskinen S, Aromaa A, et al. The impact of 29 chronic conditions on health-related quality of life: a general population survey in Finland using 15D and EQ-5D. Quality of life research : an international journal of quality of life aspects of treatment, care and rehabilitation. 2006;15(8):1403-14.

4. Kontodimopoulos N, Pappa E, Papadopoulos AA, Tountas Y, Niakas D. Comparing SF-6D and EQ-5D utilities across groups differing in health status. Quality of life research : an international journal of quality of life aspects of treatment, care and rehabilitation. 2009;18(1):87-97.

5. Cunillera O, Tresserras R, Rajmil L, Vilagut G, Brugulat P, Herdman M, et al. Discriminative capacity of the EQ-5D, SF-6D, and SF-12 as measures of health status in population health survey. Quality of life research : an international journal of quality of life aspects of treatment, care and rehabilitation. 2010;19(6):853-64.

6. Heyworth IT, Hazell ML, Linehan MF, Frank TL. How do common chronic conditions affect health-related quality of life? The British journal of general practice : the journal of the Royal College of General Practitioners. 2009;59(568):e353-8.

7. Uyl-de Groot CA, Vermorken JB, Hanna MG, Jr., Verboom P, Groot MT, Bonsel GJ, et al. Immunotherapy with autologous tumor cell-BCG vaccine in patients with colon cancer: a prospective study of medical and economic benefits. Vaccine. 2005;23(17-18):2379-87.

8. Wiering B, Oyen WJ, Adang EM, van der Sijp JR, Roumen RM, de Jong KP, et al. Long-term global quality of life in patients treated for colorectal liver metastases. The British journal of surgery. 2011;98(4):565-71; discussion 71-2.

9. Kimman ML, Dirksen CD, Lambin P, Boersma LJ. Responsiveness of the EQ-5D in breast cancer patients in their first year after treatment. Health and quality of life outcomes. 2009;7:11.

10. Whynes DK. Does the correspondence between EQ-5D health state description and VAS score vary by medical condition? Health and quality of life outcomes. 2013;11:155.

11. Dorman P, Dennis M, Sandercock P. Are the modified "simple questions" a valid and reliable measure of health related quality of life after stroke? United Kingdom Collaborators in the International Stroke Trial. Journal of neurology, neurosurgery, and psychiatry. 2000;69(4):487-93.

12. Kramer L, Hirsch O, Schlossler K, Trager S, Baum E, Donner-Banzhoff N. Associations between demographic, disease related, and treatment pathway related variables and health related quality of life in primary care patients with coronary heart disease. Health and quality of life outcomes. 2012;10:78.

13. De Smedt D, Clays E, Annemans L, Doyle F, Kotseva K, Pajak A, et al. Health related quality of life in coronary patients and its association with their cardiovascular risk profile: results from the EUROASPIRE III survey. International journal of cardiology. 2013;168(2):898-903.

14. Puumalainen A, Numminen H, Elonheimo O, Roine RO, Sintonen H. Health outcomes and costs of ischemic stroke patients in Finland. Acta neurologica Scandinavica. 2016;134(1):42-8.

15. Spiraki C, Kaitelidou D, Papakonstantinou V, Prezerakos P, Maniadakis N. Health-related quality of life measurement in patients admitted with coronary heart disease and heart failure to a cardiology department of a secondary urban hospital in Greece. Hellenic journal of cardiology : HJC = Hellenike kardiologike epitheorese. 2008;49(4):241-7.

16. Mata Cases M, Roset Gamisans M, Badia Llach X, Antonanzas Villar F, Ragel Alcazar J. [Effect of type-2 diabetes mellitus on the quality of life of patients treated at primary care consultations in Spain]. Atencion primaria. 2003;31(8):493-9.

17. Moro-Valdezate D, Buch-Villa E, Peiro S, Morales-Monsalve MD, Caballero-Garate A, Martinez-Agullo A, et al. Factors associated with health-related quality of life in a cohort of Spanish breast cancer patients. Breast cancer (Tokyo, Japan). 2014;21(4):442-52.

18. Lopez-Bastida J, Oliva Moreno J, Worbes Cerezo M, Perestelo Perez L, Serrano-Aguilar P, Monton-Alvarez F. Social and economic costs and health-related quality of life in stroke survivors in the Canary Islands, Spain. BMC health services research. 2012;12:315.

19. Staib L, Link KH, Blatz A, Beger HG. Surgery of colorectal cancer: surgical morbidity and five- and ten-year results in 2400 patients--monoinstitutional experience. World journal of surgery. 2002;26(1):59-66.

20. Geurts SM, de Vegt F, Siesling S, Flobbe K, Aben KK, van der Heiden-van der Loo M, et al. Pattern of follow-up care and early relapse detection in breast cancer patients. Breast cancer research and treatment. 2012;136(3):859-68.

21. Modrego PJ, Mainar R, Turull L. Recurrence and survival after first-ever stroke in the area of Bajo Aragon, Spain. A prospective cohort study. Journal of the neurological sciences. 2004;224(1-2):49-55.

22. Andres E, Cordero A, Magan P, Alegria E, Leon M, Luengo E, et al. Long-term mortality and hospital readmission after acute myocardial infarction: an eight-year follow-up study. Revista espanola de cardiologia (English ed). 2012;65(5):414-20.

23. Wallemacq C, Van Gaal LF, Scheen AJ. [The cost of type 2 diabetes: summary of the Cost of Diabetes in Europe-Type II study (CODE-2) and analysis of the situation in Belgium]. Revue medicale de Liege. 2005;60(5-6):278-84.

24. Pacolet J, De Coninck, A, Hedebouw, G, Cabus, S, & Spruytte, N. De medische en niet-medische kosten van kankerpatiënten. Leuven: HIVA-KU Leuven; 2011. 254 p.

25. Annemans L, Lamotte M, Clarys P, Van den Abeele E. Health economic evaluation of controlled and maintained physical exercise in the prevention of cardiovascular and other prosperity diseases. European journal of cardiovascular prevention and rehabilitation : official journal of the European Society of Cardiology, Working Groups on Epidemiology & Prevention and Cardiac Rehabilitation and Exercise Physiology. 2007;14(6):815-24.

26. Songer T, Ettaro, L, and the Economics of Diabetes Project Panel. Studies on the cost of diabetes. . Atlanta, GA: Centers for Disease Control and Prevention; 1998.

27. Kimman ML, Dirksen CD, Voogd AC, Falger P, Gijsen BC, Thuring M, et al. Economic evaluation of four follow-up strategies after curative treatment for breast cancer: results of an RCT. European journal of cancer (Oxford, England : 1990). 2011;47(8):1175-85.

28. Kanavos P, van den Aardweg, S, & Schurer, W. Diabetes expenditure, burden of disease and management in 5 EU countries. In: Medical Technology Research Group LH, London School of Economics, editor. London2012. p. 123.

29. Stock SA, Redaelli M, Wendland G, Civello D, Lauterbach KW. Diabetes--prevalence and cost of illness in Germany: a study evaluating data from the statutory health insurance in Germany. Diabetic medicine : a journal of the British Diabetic Association. 2006;23(3):299-305.

30. The national diabetes programme Dehko. Prevalence, incidence and costs of diabetes in Finland 1998-2007 2009 [Available from: https://www.diabetes.fi/en/finnish_diabetes_association/dehko/publications.

31. Farkkila N, Torvinen S, Sintonen H, Saarto T, Jarvinen H, Hanninen J, et al. Costs of colorectal cancer in different states of the disease. Acta oncologica (Stockholm, Sweden). 2015;54(4):454-62.

32. Purmonen TT, Auvinen PK, Martikainen JA. Budget impact analysis of trastuzumab in early breast cancer: a hospital district perspective. International journal of technology assessment in health care. 2010;26(2):163-9.

33. De Smedt D, Kotseva K, De Bacquer D, Wood D, De Backer G, Dallongeville J, et al. Cost-effectiveness of optimizing prevention in patients with coronary heart disease: the EUROASPIRE III health economics project. European heart journal. 2012;33(22):2865-72.

34. Athanasakis K, Ollandezos M, Angeli A, Gregoriou A, Geitona M, Kyriopoulos J. Estimating the direct cost of Type 2 diabetes in Greece: the effects of blood glucose regulation on patient cost. Diabetic medicine : a journal of the British Diabetic Association. 2010;27(6):679-84.

35. Fragoulakis V, Kourlaba G, Maniadakis N. Economic evaluation of statins in high-risk patients treated for primary and secondary prevention of cardiovascular disease in Greece. ClinicoEconomics and outcomes research : CEOR. 2012;4:135-43.

36. Carles M, Vilaprinyo E, Cots F, Gregori A, Pla R, Roman R, et al. Cost-effectiveness of early detection of breast cancer in Catalonia (Spain). BMC cancer. 2011;11:192.

37. Mata-Cases M, Casajuana M, Franch-Nadal J, Casellas A, Castell C, Vinagre I, et al. Direct medical costs attributable to type 2 diabetes mellitus: a population-based study in Catalonia, Spain. The European journal of health economics : HEPAC : health economics in prevention and care. 2016;17(8):1001-10.

38. Alvarez-Sabin J, Quintana M, Masjuan J, Oliva-Moreno J, Mar J, Gonzalez-Rojas N, et al. Economic impact of patients admitted to stroke units in Spain. The European journal of health economics : HEPAC : health economics in prevention and care. 2017;18(4):449-58.

39. Vokó Z, Nagyjanosi L, Kalo Z. Direct health care costs of diabetes mellitus in Hungary. Value in Health. 2010;13(7):A288.

40. Karpati K, Majer I, Boncz I, Nagy A, Bereczki D, Gulacsi L. [Social insurance costs of hospital treatment of stroke in Hungary, 2003-2005]. Ideggyogyaszati szemle. 2007;60(7-8):311-20.

41. Doneva M, Valov V, Borissova AM, Tankova T, Savova A, Manova M, et al. PHS87 Cost of Diabetes in Insulin-Treated Patients in Bulgaria. Value in Health. 2012;15(7):A533-A4.
